# Effects of a Multicomponent Programme for Improving Physical and Psychological Health in Victims of Intimate Partner Violence: Study Protocol for a Randomised Control Trial

**DOI:** 10.3390/ijerph20042815

**Published:** 2023-02-05

**Authors:** Violeta Calle-Guisado, Jose Carmelo Adsuar, Sabina Barrios-Fernandez, María Mendoza-Muñoz, Laura Muñoz-Bermejo, Francisco Javier Domínguez-Muñoz, Luis Ortiz-González, Jorge Rojo-Ramos

**Affiliations:** 1 Faculty of Medicine and Health Sciences, University of Extremadura, 06006 Badajoz, Spain; 2 Research Group on Physical and Health Literacy and Health-Related Quality of Life (PHYQOL), Faculty of Sport Sciences, University of Extremadura, 10003 Caceres, Spain; 3 Promoting a Healthy Society Research Group (PHeSO), Faculty of Sport Sciences, University of Extremadura, 10003 Caceres, Spain; 4 Occupation, Participation, Sustainability and Quality of Life (Ability Research Group), Nursing and Occupational Therapy College, University of Extremadura, 10003 Caceres, Spain; 5 Departamento de Desporto e Saúde, Escola de Saúde e Desenvolvimento Humano, Universidade de Évora, 7004-516 Évora, Portugal; 6 Social Impact and Innovation in Health (InHEALTH), University Centre of Mérida, University of Extremadura, 06800 Mérida, Spain; 7 Physical Activity and Quality of Life (AFYCAV) Research Group, Faculty of Sport Science, University of Extremadura, 10003 Caceres, Spain; 8 Physical Activity for Education, Performance and Health, Faculty of Sport Sciences, University of Extremadura, 10003 Caceres, Spain

**Keywords:** physical activity, IPV, psychological therapy, hormonal stress-related

## Abstract

Intimate partner violence (IPV) is a public health problem that psychologically and physically affects the women who suffer from it. In this article, we propose an intervention protocol based on therapeutic tourism through adventure physical activities and psychological therapy that could improve the psychological and physical health of women participants. We propose a randomized study where participants will be separated into two groups, control and experimental, and we will perform measurements including self-concept, self-image, depression, and perceived stress, combining these with physiological measurements of stress level by determining stress-related hormones (Cortisol and DHEA), as well as the overall cost-effectiveness of the program. All data collected at the end of the protocol will be statistically analysed. If the final data are positive and it is feasible, this protocol could be proposed as a measure for the treatment of the sequelae of victims of gender violence.

## 1. Introduction

Currently, there is a growing concern about health and well-being-related topics, triggered by the global pandemic we experienced since March 2020. Therefore, the 2030 Agenda developed by the United Nations (UN) has included health and well-being among the Sustainable Development Goals (SDGs) [1]. Hence, within SDG-3, Health and Well-being, mental health promotion has a privileged place, due to the alarming increase in mental health problems, and warning the World Health Organisation (WHO) that by 2030 these disorders will be the leading cause of disability in the world [2].

Therapeutic tourism brings together both the WHO definition of health tourism and the World Tourism Organisation (UNWTO) definition as an activity that involves a person travelling to a place other than their place of residence to receive a health intervention through tourist services, products, or experiences that the destination promotes and offers González, et al. [3]. There are several approaches within this therapeutic tourism including, on the one hand, the medical-clinical, focused on pathology, aesthetic or reproductive issues, and on the other hand, wellness tourism, which impacts overall health González, González and Hernández [3]. This project will focus on the second one, as the tourism motivation will (1) improve or maintain physical health and appearance, (2) improve or maintain mental health, (3) restore self-esteem, (4) the importance of others, (5) self-discovery, (6) personal development, (7) transformation of oneself and (8) specialisation of the destination in a specific therapy or service [3,4]. The Jerte Valley is one of the most well-known and consolidated tourist destinations in Extremadura, positioned nationally as one of the most popular destinations in rural and nature tourism. Due to its orography, it offers optimal conditions for the practice of various physical activities in nature and adventure sports, hosting more than 70% of the Active Tourism companies in Extremadura [5]. Consequently, the Jerte Valley natural environment has the proper attributes to become a successful therapeutic tourism destination, given as its characteristics include tourist-resident interaction in the tourist destination, natural landscape, suitable climate and landscape as a whole, pollution-free location, the existence of a suitable therapeutic tourism offer, safety in general and health safety in particular [3,6,7,8,9].

The UN defines violence against women (VAW) as any act of gender-based violence that results in or is likely of resulting in, physical, sexual, or mental harm or suffering to women, including threats of such acts, coercion, or arbitrary deprivation of liberty, whether occurring in public or private life and may include different forms of violence such as intimate partner violence (IPV), sexual violence, human trafficking, female genital mutilation, or child marriage [10,11,12]. IPV is committed by a current or former intimate partner and is considered the most common form of violence experienced by women, resulting in physical, psychological, and sexual violence or stalking; one in three women in a relationship has experienced IPV, according to the UN [13]. Gender violence has serious consequences for women’s health, particularly post-traumatic stress disorder (PTSD) as a consequence of the suffered violence [14,15]. Women, as a consequence of IPV, have a damaged body image which leads to a negative self-concept [16] and aggravates the PTSD symptomatology [17]. Moreover, PTSD impacts people’s well-being; in the case of IPV victims, affecting their well-being and physiological responses [18,19]. Several studies have analysed the endocrine and immune consequences of IPV, revealing disturbances in the pituitary-adrenal axis functioning of the hypothalamus [20]. It has also been found that IPV victims who developed PTSD have elevated levels of cortisol during the day, with is associated with this hormone production dysregulation leading to physical stress symptoms and other associated pathologies [21]. IPV victims also have higher nocturnal cortisol levels of dehydroepiandrosterone (DHEA) compared to women who have not been IPV victims [13].

The connection with nature can be an alternative approach for people with psychological problems [22]. Nature therapy combines several techniques that address factors in the origin and maintenance of psychosocial problems, making it more effective than individual techniques. Studies have demonstrated its many beneficial features, such as its focus on direct user participation and responsibility, its interaction with the natural environment, and its attention to problem-solving [23]. Moreover, the American Psychological Association (APA) recommends its use for IPV victims [24]. To date, a protocol for a multicomponent cognitive-behavioural program based on therapeutic tourism has been published [25]. However, no measurements of physiological aspects were carried out in this study. Then, as therapeutic tourism has suitable properties to work with IPV victims [26] and to advance knowledge in this field, this study will aim to analyse the effects of a multicomponent program combining psychological therapy and adventure sportive activities in the Jerte Valley to improve the general well-being and health of IPV victims, assessing biological markers of stress and aspects related to mental health.

## 2. Materials and Methods

### 2.1. Study Design and Ethical Considerations

A parallel-group randomized controlled trial will be conducted including a one-month intervention phase. Both for the control group (CG) and experimental group (EG), evaluations will be carried out before the start of the intervention (initial assessment), as well as at the end of the intervention (final assessment). The study will be carried out according to the Consolidated Standards of Reporting Trials Statement (CONSORT) methodology for randomized controlled trials [27].

The Ethics Committee of the University of Extremadura University approved this project (approval code: 45/2022), which will follow the Declaration of Helsinki and their actualizations as amended by the 64th General Assembly of the World Medical Association (Fortaleza, Brazil, 2013) and the Biomedical Research Act 14/2007. Moreover, this study has been registered with the International Standard Randomised Controlled Trial Number (registration number: ISRCTN93835057; https://www.isrctn.com/) (accessed 26 October 2022).

### 2.2. Sample Size

Assuming a dropout rate, and accepting an alpha risk of 0.05 and a beta risk of 0.2 in a two-sided test, 11 subjects should be necessary for the intervention and 11 in the control group to recognize, as statistically significant, a difference greater than or equal to 0.18 units [28]. The common standard deviation was assumed to be 0.15 [29], and the correlation coefficient between the initial and final measurement, was 0.6 [30].

### 2.3. Randomisation and Blinding

Participants will be randomized 1:1 to the intervention (usual care + multi-component plan) or the control group (usual care). A research team member who will not be directly involved in the trial will create a simple computer-generated randomization sequence using the software Research Randomizer (version 4.0; http://www.randomizer.org, accessed on 20 April 2022) [31]. The assignment will be hidden with a password-protected file. The researchers involved in the data analysis processes will not be aware of the group to which each woman will be assigned (EG or CG).

### 2.4. Participants

This study will be carried out in Caceres (Extremadura, Spain), and the participants will be women who have suffered from IPV. For the participants’ recruitment, information leaflets will be developed and distributed among different social resources for IPV victims in the Community of Extremadura. For this purpose, social workers from different IPV victim care resources and people belonging to associations with direct contact with victims will invite the participants on a completely voluntary basis. The participants will be asked for a contact form (email or a telephone number created for this purpose) so that the psychologist of the project can get in touch with them to carry out the evaluation and for eligibility criteria verification. All participants will provide a signed informed consent form agreeing to participate in the study.

Participants will have to meet the following inclusion criteria: (1) to be a woman; (2) over 18 years of age; (3) have suffered IPV, and (4) to be residents in Extremadura. The exclusion criteria include (1) comorbidity with other psychological diseases such as severe mental disorder (schizophrenia or bipolar disorders), personality or eating disorders such as anorexia or bulimia, as these psychological problems require interventions more specific to their nature; (2) participants with comorbidity of physical problems that prevent them from carrying out adventure activities in nature and (3) participants who cannot speak the Spanish language enough to hold a conversation.

### 2.5. Intervention

**Figure 1:**
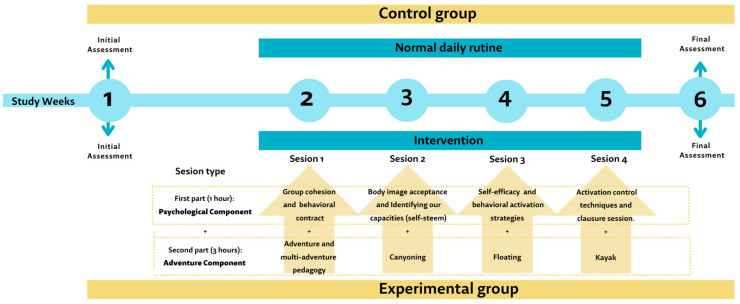
Intervention diagram.

EG: A multicomponent cognitive-behavioural four-week program (one session per week) will be carried out. This program will be developed over four weekends and will consist of outings to the Jerte Valley for participation both in therapeutic tourism through adventure physical activities and psychological therapy, so every session will be divided into two parts: the psychological component with a one-hour session of group therapy on different psychological and social aspects, conducted by clinical psychologists specializing in IPV and will prepare the women for the adventure activity; and a second part which will consist of different adventure activities, conducted by sport sciences professionals, with an approximate duration of three hours (Figure 1). If necessary, children may accompany their mothers during the intervention. These will be cared for by specialized monitors so that their mothers can carry out the planned activities.

CG: will continue with their usual care.

### 2.6. Measures and Instruments

Various instruments will be used to assess the feasibility and effectiveness of the multi-component program. Evaluation will be carried out in both EG and CG at baseline and post-intervention.

Self-reported psychological assessment will include the following instruments in a paper format booklet:

- Brief socio-demographic data sheet: this will include questions related to age, gender, educational level and socio-economic status, etc., and gender-based violence history (questions to assess lived experiences of gender-based violence).
- Baessler and Schwarzer’s General Self-Efficacy Scale [32]. It aims to assess a stable sense of personal competence to deal effectively with multiple stressful situations. This questionnaire is composed of 10 items with a 4-point Likert-type response.
- Rosenberg Self-Esteem Scale [33]. The scale assesses feelings of self-respect and self-acceptance. The scale includes ten items, half of which are stated positively and half negatively. The items are answered on a 4-point Likert scale (1 = strongly agree, 2 = agree, 3 = disagree, 4 = strongly disagree), and the total score ranges from 10 to 40. Participants will complete the Spanish version adapted by Morejón, et al. [34] (α= 0.72 to 0.88).
- Body Shape Questionnaire (BSQ) [35]. This questionnaire assesses the presence of concern and dissatisfaction with body image. It measures dissatisfaction with one’s own body, fear of gaining weight, low self-concept about physical appearance, and desire to lose weight. It consists of 34 items including 4 subscales (body dissatisfaction, fear of gaining weight, low esteem related to appearance, and desire to lose weight) which are measured on a 6-point scale, ranging from “never” to “always”. The total score ranges from 34 to 204, with higher scores showing greater body dissatisfaction. The Spanish validation of Raich, et al. [36], will be completed by participants (α = 0.95 to 0.97) [36].
- Beck Depression Inventory, Second Edition (BDI-II) [37]. This is a questionnaire that assesses the presence and severity of depression in adolescents and adults. This consists of 21 items from different areas such as sadness, loss of pleasure, suicidal ideation, etc. Scores range from 0 to 63, including different cut-off points to classify patients according to the following subgroups: normal to minimal depression (0 to 13), mild depression (20 to 28), moderate depression (20 to 28), and severe depression (scores above 29) (α = 0.83) [38].
- Perceived Stress Test (PSS) [39]. The PSS assesses the perception of control over environmental demands. It is a self-report instrument consisting of 14 items with a five-point scale response format (0 = never, 1 = hardly ever, 2 = occasionally, 3 = often, 4 = very often). A higher total score obtained is an indication of a higher level of perceived stress (α = 0.82 to 0.81) [40].
- EQ-5D-5L. The EQ-5D-5L [41] is a questionnaire that aims to assess health-related quality of life (HRQoL). It is composed of 5 dimensions (mobility, self-care, usual activities, pain or discomfort, and anxiety or depression), with five possible response levels. Health states can be described by a 5-digit number, where the first number is the response to the first question, the second number is the response to the second question, and the third number is the response to the third question. is the answer to the first question, the second number is the answer to the second question, and so on. Given Since the EQ-5D is a preference-based questionnaire, these health states can be converted into a utility index by applying the appropriate formula. This instrument also includes a Visual Analogue Scale (VAS) with a range from 0 (worst imaginable health state) to 100 (best imaginable health state). (best imaginable health state).
- Physiological measures will include glucocorticoids, cortisol, and dehydroepiandrosterone (DHEA) analysis through saliva samples. The participants will provide a minimum of 0.5 mL of saliva in a plastic tube recipient Salitubes SLV-4157 (DRG Instruments GmbH, Marburg, Germany) specifically to be used with DRG Salivary Cortisol/DHEA ELISA Kits (DRG Instruments GmbH, Marburg, Germany) twice a day (between 8 and 9 AM for DHEA and cortisol, and between 8 and 9 PM for evening cortisol). All samples will be triplicated to ensure process reliability. Participants will be instructed to collect samples in their homes. Saliva samples will be frozen in women’s freezers and will be brought to the Department of Anatomy of the University of Extremadura medicine Faculty in a mobile freezer, where they were being kept frozen at 21 °C previously to be analysed following the protocol indicated in the kits.

### 2.7. Cost-Effectiveness Analysis

A cost-effectiveness analysis will be conducted with a health system perspective in line with the methodological recommendations of health economists [42], and the guidelines defined in the scientific literature [43]. The study will be conducted considering the costs and health effects of the intervention. Only the direct costs of the program will be considered. Direct costs include medication, primary care visits, and hospital admissions. The salaries of the staff hired under the project will also be considered. It also will include the material costs necessary for the combined intervention.

The average costs and effectiveness, i.e., Quality Adjusted Life Years (QALYs) gained, will be calculated for each group. Subsequently, the incremental cost-effectiveness ratio will be calculated [44], and various sensitivity analyses will be performed, including probabilistic analysis, which will be carried out with 1000 replicates [45]. These replicates will be included in the cost-effectiveness plane and will be used to make the acceptability curve. After doing this we will be able to see in which quadrant of the cost-effectiveness plane the program falls, which will indicate whether the intervention has been more or less costly and effective than the one carried out by the CG.

### 2.8. Statistical Analysis

All data and analyses will be collected and analysed with the SPSS statistical package (version 26.0; SPSS, Inc., Chicago, IL, USA). The Shapiro-Wilk test will be performed to check data normality and to choose between parametric or non-parametric statistical analyses. Continuous socio-demographic variables will be presented as mean (SD) or median (IR) where appropriate, while categorical variables will be presented as proportions. Differences between groups at baseline will be analysed using independent samples t-tests. To assess the effects of the program, two types of analyses will be conducted. Intervention effects will be assessed using a repeated measures ANCOVA test, adjusted for age and baseline values. Cohen’s d (with a 95% confidence interval) will be also included in the results as the effect size. Effect size thresholds will be interpreted as follow: >0.2, small; >0.5, moderate; >0.8, large [46]. Statistical significance will be computed for the effect of time and the interaction group × time. The alpha level will be fixed at *p* ≤ 0.05.

## 3. Discussion

This project would be the first to combine psychological therapy with adventure activities in nature, measuring psychological aspects and taking physiological measurements of biological markers of stress to improve the general well-being and health of women victims of gender-based violence. To our knowledge, it will be a pioneer in analyzing the physiological and psychological effects of a multi-component psychosocial program in a natural environment.

Activities in nature seem to have a significant potential to provide a suitable environment to gain self-awareness, relax, connect with others, and promote self-care [47]. In addition, natural environment interventions have great potential to alleviate stress and support the recovery process of victims [48]. A physiological improvement in stress levels through cortisol measurements would demonstrate that psychological intervention together with therapeutic tourism activities would reduce stress-related illnesses such as trauma and PTSD. Creating an empowering wilderness experience for these women could help them acquire and develop emotional competencies to cope with life change; adventure activities in combination with psychological therapy could also help women learn about their coping capacities in unfamiliar situations [49,50,51,52]. Psychological therapy could reaffirm their self-concept and self-esteem, improving their emotional well-being [14,53,54].

Although one of the main limitations may be that the program has a short duration, IPV women may have childcare issues or other that may cause them to drop out of the intervention [55], so it was considered that four sessions may be reasonable. We will also include child care in the protocol to prevent this from being a problem or abandoning the intervention. Staying out overnight is also avoided so as not to compromise the availability of the participants. We are aware of the difficulty of accessing and performing this intervention with this type of vulnerable population. However, we believe that these limitations make this proposal original and novel. Based on the obtained results, future studies will address possible limitations.

## 4. Conclusions

If positive results on women’s emotional well-being are obtained, and if it is a cost-effective type of intervention, the implementation of multi-component programmes using the resources of therapeutic tourism could be a tool to empower and support the mental health of these women, as well as optimising costs for the social and health systems.

## Data Availability

Not applicable.
